# Strain and Electromyography Dual-Mode Stretchable Sensor for Real-Time Monitoring of Joint Movement

**DOI:** 10.3390/mi17010077

**Published:** 2026-01-06

**Authors:** Hanfei Li, Xiaomeng Zhou, Shouwei Yue, Qiong Tian, Qingsong Li, Jianhong Gong, Yong Yang, Fei Han, Hui Wei, Zhiyuan Liu, Yang Zhao

**Affiliations:** 1 School of Airspace Science and Engineering, Shandong University, Weihai 264209, China; hf.li2@siat.ac.cn (H.L.); gongjh@sdu.edu.cn (J.G.); 2 Neural Engineering Centre, Shenzhen Institute of Advanced Technology, Chinese Academy of Sciences, Shenzhen 518055, China; zhouxm@siat.ac.cn (X.Z.); qiong.tian@siat.ac.cn (Q.T.); qs.li@siat.ac.cn (Q.L.); fei.han@siat.ac.cn (F.H.); 3 Weihai Research Institute of Industrial Technology, Shandong University, Weihai 264209, China; 4 Department of Physical Medicine & Rehabilitation, Qilu Hospital, Shandong University, Jinan 250012, China; shouweiy@sdu.edu.cn (S.Y.); kkkk-9806@163.com (H.W.); 5 Hand Surgery Department, Beijing Jishuitan Hospital, Capital Medical University, Beijing 100035, China; 0414yang@sina.com

**Keywords:** stretchable electronics, SEBS, nanogold film, capacitive strain sensor, electromyography monitoring, dual-mode

## Abstract

Flexible sensors have emerged as critical interfaces for information exchange between soft biological tissues and machines. Here, we present a dual-mode stretchable sensor system capable of synchronous strain and electromyography (EMG) signal detection, integrated with wireless WIFI transmission for real-time joint movement monitoring. The system consists of two key components: (1) A multi-channel gel electrode array for high-fidelity EMG signal acquisition from target muscle groups, and (2) a novel capacitive strain sensor made of stretchable micro-cracked gold film based on Styrene Ethylene Butylene Styrene (SEBS) that exhibits exceptional performance, including >80% stretchability, >4000-cycle durability, and fast response time (<100 ms). The strain sensor demonstrates position-independent measurement accuracy, enabling robust joint angle detection regardless of placement variations. Through synchronized mechanical deformation and electrophysiological monitoring, this platform provides comprehensive movement quantification, with data visualization interfaces compatible with mobile and desktop applications. The proposed technology establishes a generalizable framework for multimodal biosensing in human motion analysis, robotics, and human–machine interaction systems.

## 1. Introduction

Accurate monitoring of joint movement is critical for applications ranging from sports science to human–machine interfaces, requiring simultaneous measurement of mechanical deformation and muscle activation patterns [1,2,3,4,5,6,7]. Traditional rigid sensors struggle to track complex joint kinematics due to mechanical mismatch with biological tissues and an inability to capture multimodal physiological signals. These challenges have spurred the development of flexible hybrid sensing systems that integrate strain and electromyography (EMG) measurements for comprehensive motion analysis. Recent advances in flexible electronics have enabled new approaches to joint monitoring through synergistic sensor fusion. Strain sensors can quantify joint angular displacement via surface deformation mapping, while synchronized EMG signals provide complementary information about muscle recruitment patterns. However, existing systems often suffer from three key limitations: (i) strain measurement artifacts caused by sensor placement variations on curved joints, (ii) temporal desynchronization between mechanical and electrophysiological signals during dynamic movements, and (iii) a lack of robust wireless systems for real-time monitoring outside laboratory settings.

**Figure 1:**
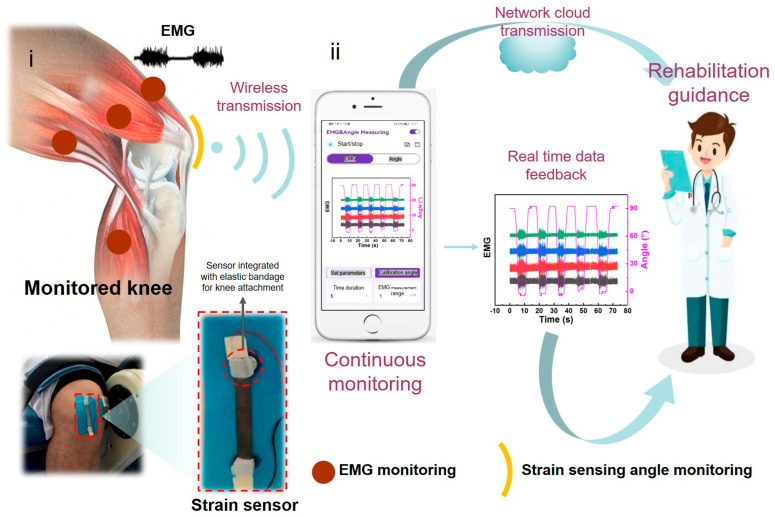
Schematic diagram of strain and electromyography dual-mode stretchable sensor for real-time monitoring of joint movement, including (**i**) fabricated dual-mode sensor (strain sensor + EMG electrode array) and (**ii**) dual-mode monitoring data program display.

This project plans to develop a strain–EMG dual-mode flexible sensor for continuous real-time monitoring to monitor the bending angle of the joint, skin stress, and strain caused by joint swelling. It aims to cooperate with multi-channel electromyography electrodes to achieve real-time monitoring of muscle electrical signals [8,9,10,11,12,13,14,15,16], continuously monitor and analyze the status of joints, provide real-time guidance for the joint activity and weight bearing, and develop wireless WIFI signal transmission module and corresponding signal acquisition and analysis software to provide the basis for doctors’ remote diagnosis and treatment in the future (Figure 1 and Figure S1). It is conducive to scientific, systematic, personalized, and professional treatment of joint movement rehabilitation [17].

The synchronized strain and EMG signals directly map to core rehabilitation metrics. For joint rehabilitation (e.g., post-ligament injury), the strain-derived knee angle quantifies the range of motion—a key indicator of tissue healing and functional recovery. Deviations from clinically recommended range-of-motion thresholds (e.g., 0–120° for post-ACL reconstruction) trigger real-time alerts for dosage adjustment. Meanwhile, EMG signals reflect muscle activation patterns: abnormal amplitude fluctuations indicate compensatory movements or muscle weakness, which are critical for personalizing exercise intensity and correcting movement biomechanics. By integrating these metrics, the system enables the objective assessment of rehabilitation progress and provides data-driven guidance to avoid overloading or underutilization of the injured joint—addressing the unmet need for quantitative, personalized rehabilitation monitoring.

At present, there has been a lot of research on the preparation methods of flexible and stretchable sensors, and many strategies have been put forward in the international community to solve the problem of how to realize the design of a coating structure that can still conduct electricity under great levels of deformation [18,19,20,21,22,23]. The core component of a flexible stretchable thin film sensor is a coating structure that can still conduct electricity under strong deformation, because the general metal and non-metal conductive film will produce significant penetrating cracks under any more than 10% strain, leading to film failure. Therefore, how to maintain a conductive path under strains of up to 100% or even higher is a huge challenge [24,25,26,27,28,29,30,31,32]. The performance optimization of flexible strain sensors essentially stems from the deep synergy between the physical and chemical properties of conductive materials and flexible substrates, which construct the core functional framework of the sensor from two dimensions: the electrical response mechanism and mechanical deformation adaptation [33]. As the electrical carrier of the sensing unit, conductive materials generate predictable resistance/capacitance changes during deformation through their microstructure (such as nano-networks, ion channels, or liquid-metal droplets). The conductivity type (electronic conductivity, ion conductivity, or mixed conductivity), leakage threshold, and dynamic stability directly determine the sensitivity, linearity, and hysteresis characteristics of the sensor. For example, silver nanowire networks rely on overlapping point slip to achieve continuous conductive pathways under high strain [34]. At the same time, as a mechanical support platform, flexible substrates need to match human tissue or target carriers in parameters such as elastic modulus (usually 0.1 kPa–10 MPa), elongation at break (>100%), and fatigue durability (>10^4^ cycles), and convert external mechanical loads into controllable, uniform deformation through energy dissipation mechanisms such as molecular chain disentanglement and micro-crack deflection. PDMS and other silicon-based elastomers achieve modulus gradient design by adjusting the degree of crosslinking, while the hydrogel substrate balances flexibility and tear resistance with the help of a dual-network structure. The synergistic effect of the two is reflected in the dynamic adaptation of the deformation response mechanism of conductive materials to the stress–strain transfer characteristics of the substrate; when the substrate undergoes tension/compression, the internal stress field distribution needs to guide the conductive network to undergo ordered structural evolution (such as nanowire orientation and graphene interlayer slip), rather than disordered fracture. A typical example of this collaborative optimization includes constructing a silver nanowire/graphene hybrid conductive layer on a pre-stretched 300% Ecoflex substrate, and releasing the pre-strain to form a multilevel wrinkled structure that can maintain the sensor’s ΔR/R_0_ < 8% at 400% working strain [35]. By adopting a modulus-matching strategy (such as matching the compression modulus of MXene conductive ink (≈1.2 MPa) with PU sponge substrate (≈0.8 MPa)), the interfacial shear stress can be significantly reduced, resulting in a resistance drift rate of less than 3% after 10,000 50% strain cycles of the device. Beyond joint rehabilitation, multimodal stretchable sensors have broad cross-disciplinary applications. For example, machine learning-enhanced soft robotic systems inspired by rectal functions have been developed to investigate fecal incontinence [36], where synchronized biomechanical and electrophysiological sensing is critical for understanding tissue–machine interactions. Similarly, our dual-mode system (strain + EMG) provides a generalizable framework for such cross-disciplinary applications, enabling quantitative analysis of muscle–joint coordination in both human health monitoring and soft robotics.

Herein, we have developed a thin-film strain sensor based on a micro-crack gold film stretching mechanism, which can be used in conjunction with motion bandages and wireless transmission devices to provide real-time monitoring of and feedback on human joints. This work focuses on a capacitive strain sensor design, where mechanical deformation (e.g., joint bending-induced stretching) modulates the capacitance of an SEBS-based micro-cracked gold film structure. Capacitive sensing relies on changes in electrode overlapping area, dielectric thickness, or permittivity under strain—offering the advantages of low energy consumption, high linearity, and minimal signal drift compared to resistive or piezoelectric alternatives, making it ideal for long-term wearable joint monitoring. This capacitive sensor has excellent tensile properties (>80%), cycle performance (>4000 times), linearity, and response time (<100 ms).

## 2. Experiments and Methods

The flexible stretchable sensor is light, transparent, and easy to attach to the skin. Unlike the traditional sensor, the elastic substrate cannot use hard and brittle materials such as glass and ceramics, but instead uses high-molecular-organic-polymer materials such as graphene, polydimethylsiloxane (PDMS), polyethylene film (PET), polyimide (PI), and polyurethane (PU). From the many flexible materials, Styrene Ethylene Butylene Styrene (SEBS) was finally selected as the flexible substrate. Unlike unsaturated elastomers (e.g., natural rubber), SEBS lacks carbon–carbon double bonds in its backbone, which eliminates oxidative cross-linking or chain scission under repeated mechanical stress and environmental exposure. This chemical stability directly enhances the sensor’s long-term durability: the absence of reactive double bonds prevents material aging (e.g., brittleness, yellowing), ensuring reliable capacitance–strain response over prolonged use. Additionally, this structural feature improves the adhesion stability of the sputtered gold film, reducing interfacial delamination during cyclic stretching—critical for preserving the conductive-network integrity of the micro-cracked electrode. The flexible electrode materials and backing generally include metal films, ionic conductors, metal nanowires, ITO, pet, and experimental sections.

**Figure 2:**
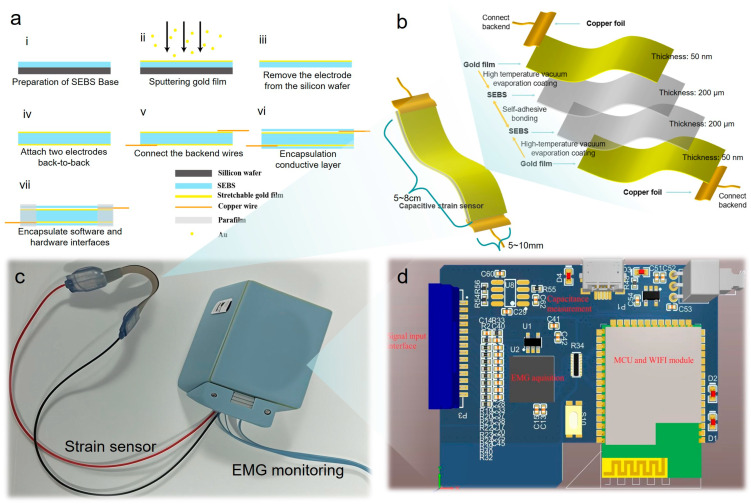
(**a**) Fabrication process diagram of capacitive strain sensor. (**i**) Spin coating of SEBS on a silicon wafer, (**ii**) deposition of the gold film by magnetic sputtering, (**iii**) taking the electrode off the silicon wafer and cutting it into slices, (**iv**) the combination of two pieces back-to-back through the self-adhesion of SEBS to form the capacitance, (**v**) connecting copper wires to both sides of the capacitance, (**vi**) encapsulation of the capacitance sensor by SEBS insulation layers, (**vii**) encapsulation of the capacitance sensor by parafilm layers. (**b**) Schematic diagram of disassembly of sensors and their various parts. (**c**) Schematic diagram of strain and electromyography dual-mode wireless acquisition device. (**d**) Schematic diagram of internal electronic circuit of wireless device, including EMG Signal Acquisition Module (ADS1298), Capacitance–Frequency Conversion Module (LMC555), and “Data Processing Unit (MCU), Wireless Transmission Module. The circuit integrates two signal paths: (1) EMG signals are amplified and digitized by the ADS1298 chip (Shenzhen, China); (2) strain-induced capacitance changes are converted to frequency via the LMC555 RC oscillator (Shenzhen, China).

To fabricate the stretchable strain sensor, firstly, SEBS was diluted in toluene with a weight concentration of 15% and then dropped onto a silicon wafer manually. Subsequently, the SEBS was cured at 25 °C for 24 h. As a result, an SEBS coating with 100 μm thickness was prepared. A magnetic sputtering system (JS4S-75G, Beijing, China) was adopted to deposit a gold film of 25 nm thickness over 15 s. The stretchable gold film electrodes were obtained after peeling off the mask at the end of the sputtering process. Then the stretchable electrodes were cropped into slices of the proper size. After that two pieces of the stretchable electrode were combined together by attaching them by the back sides, taking advantage of the characteristic of self-adhesion of SEBS to compose a capacitance sensor. By measuring the thickness uniformity of the self-adhesive layer through an optical microscope, it can be seen that the thickness of the dielectric layer remains almost unchanged at different positions of the same sample (Figure S2), indicating that the back-to-back self-adhesive method is stable and reliable. Later on, copper wires were connected to two ends of the sensor. Finally, the sensor was encapsulated with an SEBS layer and parafilm to keep it insulated and robust. The fabrication processes are illustrated in Figure 2a. The complete sketch map of the device and its corresponding disassembly diagram are shown in Figure 2b. The physical image of the sensor is shown in Figure S3. The schematic diagram of the entire dual-mode device is shown in Figure 2c, and its internal working principle, related electronic circuits, and various modules are shown in Figure 2d. Statistical comparison shows that the thickness uniformity of the electrodes between different batches is very good, and the thickness is basically maintained at 360–370 μm (Figure S4).

## 3. Results and Discussions

The flexible capacitive strain sensor converts external mechanical deformation into electrical signals through capacitance modulation. The sensor consists of two parallel conductive gold film electrodes with an SEBS dielectric layer in between. When subjected to tensile strain (e.g., joint bending), the SEBS substrate undergoes axial elongation and lateral contraction (governed by Poisson’s ratio), which simultaneously increases the effective overlapping area (A_0_) of the electrodes and decreases the dielectric layer thickness (d_0_). These changes alter the capacitance value (Equation (1)) in a predictable manner, enabling the quantitative measurement of strain and derived joint angles.

According to the working mechanism, the traditional sensors are piezoelectric, resistive, capacitive, and triboelectric. In contrast, the resistance sensor will generate heat due to resistance change during measurement, which is sensitive to temperature and has general accuracy; piezoelectric sensors can only measure static pressure signals and have a high-frequency response. They should be used together with charge amplifiers. In general, capacitive sensors are easy to realize, simple to operate, and have low requirements of equipment for processing and design. The linearity under pure stretching is better than that of resistive sensors. In general, it is not easy to produce signal drift, high response repeatability, and low energy consumption in the process, which are more suitable for application on the changeable human body. The theoretical model of the capacitive, flexible, and stretchable pressure sensor is shown in Figure 2. It is composed of two layers of flexible conductive materials and an elastic substrate in the middle. When the element is stimulated by external pressure, the charge changes, and thus the capacitance changes. The capacitance changes are affected by the area s of the upper and lower layers of electrodes and the dielectric layer, the distance d between the plates, and the relative dielectric constant of the dielectric layer εr. The capacitance space absolute dielectric constant is ε_0_. If the field side effects of the capacitor plate are ignored, the capacitance calculation formula can be expressed:

(1) $$C_{0}=\varepsilon_{0}\varepsilon_{r}\frac{{A_{0}(1+\epsilon )}^{2}}{d_{0}(1+\nu \epsilon )}$$

*C*: *Capacitance value*

*ε*_0_: *Vacuum dielectric constant*

*ε_r_*: *Relative dielectric constant of dielectric materials*

*A*_0_: *Initial effective overlapping area of electrodes*

*d*_0_: *Initial thickness of dielectric layer*

ν : *Poisson’s ratio*

*ϵ*: *strainv*

The change in capacitance value is mainly determined by the distance between the plates and the dielectric constant. The characteristics of the flexible electrodes and elastic substrates of different materials are important factors affecting the capacitance value. Therefore, capacitive flexible sensors with different energy can be fabricated by designing different sizes and applying different flexible conductive materials and elastic substrates.

**Figure 3:**
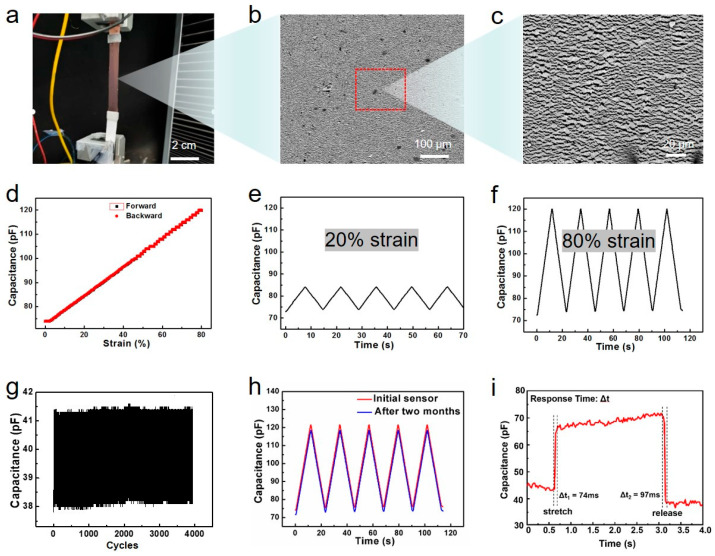
Performance characterization data of strain sensors. (**a**) The physical image of the sensor on the stretching machine. (**b**,**c**) SEM images of the sensor electrode surface. (**d**) Performance comparison of the device during stretching and contraction. (**e**,**f**) Multiple stretching data of the sensor under different deformations. (**g**) Stretching cycle performance of the sensor. (**h**) Performance change in the sensor after long-term placement. (**i**) Response time data of the sensor.

The stretchable sensor is made of a stretchable gold film deposited on the elastic SEBS substrate. The mechanical properties of the sensors are characterized by stretching measurements in a universal mechanical tester, shown in Figure 3. As shown in Figure 3a–c, when the sensor is stretched, numerous micro-cracks are generated on the electrode surface to release stress and ensure the continuity of the entire gold film to maintain the electrical performance of the device. This is due to its inherent micro-crack structure (Figure S5): (1) The cracks are microscale and non-penetrating—formed via controlled strain during sensor fabrication, they act as stress release channels rather than complete conductive pathway disruptors. The remaining gold film segments between cracks form a continuous ‘island–bridge’ network, preserving electronic conductivity even at 80% strain. (2) The SEBS substrate’s high elasticity ensures crack closure during relaxation, minimizing contact resistance between gold film segments. SEM imaging confirmed that all of the Au film area formed uniform micro-cracks (width: 20–50 nm, spacing: 100–300 μm) across different batches (Figure S6). No batch showed excessive crack width (>1 μm) or crack-free regions, indicating a high yield of the micro-crack structure. In addition, we evenly divided a 5 μm × 5 μm image into 25 parts and manually counted the number of micro-cracks in each area. It can be seen that there are 4–9 micro-cracks evenly distributed in each square micrometer area (Figure S7). Based on the statistical results of all areas, the density of micro-cracks is calculated to be 5.52/μm^2^. These statistical data confirm the uniformity of the micro-crack network, with smaller crack widths and a larger number of cracks preventing local stress concentration, ensuring the continuous conductivity of the Au film during the tensile process. From Figure 3d–f, it can be seen that the deformation capacitance curve of the device has high linearity (R^2^ > 0.99) and negligible hysteresis (<2%) phenomena during the stretching and release process under different deformations (unlike resistance strain sensors), which is ideal for sensor applications. In addition. Figure 3g indicates that the sensor has a cyclic stability of over 4000 strain cycles. Figure 3h shows that the electrode performance does not decrease with retirement over time, and all of these properties are beneficial for its long-term storage and repeated use. Finally, the response time of the sensor was tested, and its stretching and releasing response times were both between 70 and 100 ms (Figure 3i), which meet the requirements.

Unlike conventional crack-based sensors (e.g., spider-inspired crack sensors [5], graphene crack sensors [3]), our micro-cracked gold film on SEBS exhibits unique structural advantages that improve key performance metrics: traditional crack-based sensors rely on irreversible crack propagation, leading to hysteresis > 10% [5]. Our micro-cracks are pre-formed and reversible—SEBS’s high elasticity (recovery rate > 99%) closes cracks completely during relaxation, eliminating residual deformation. This results in hysteresis <2% (Figure 3d), which is significantly lower than reported for crack-based sensors (hysteresis: 8–15% [3,5]). The uniform micro-crack distribution ensures that strain is evenly distributed across the electrode, avoiding localized stress concentration. This leads to a linear capacitance–strain response (R^2^ > 0.99, Figure 3e,f), whereas conventional crack-based sensors show non-linear behavior (R^2^ < 0.95) due to random crack formation [34]. The accelerated aging test was conducted using the principle of temperature equivalence in a high-temperature oven. A batch of devices were placed in a 60 °C oven for 7 days (equivalent to 9–12 weeks at room temperature), and their capacitance changes were tested every two days. From the graph, it can be seen that their capacitance did not increase or decrease significantly with aging time, proving the stability of the devices over time. In addition, we also conducted tensile cycling tests at a temperature of 50 degrees. The capacitance change in the device tends to stabilize before and after 200 cycles of tensile testing at high temperatures (Figure S8).

**Figure 4:**
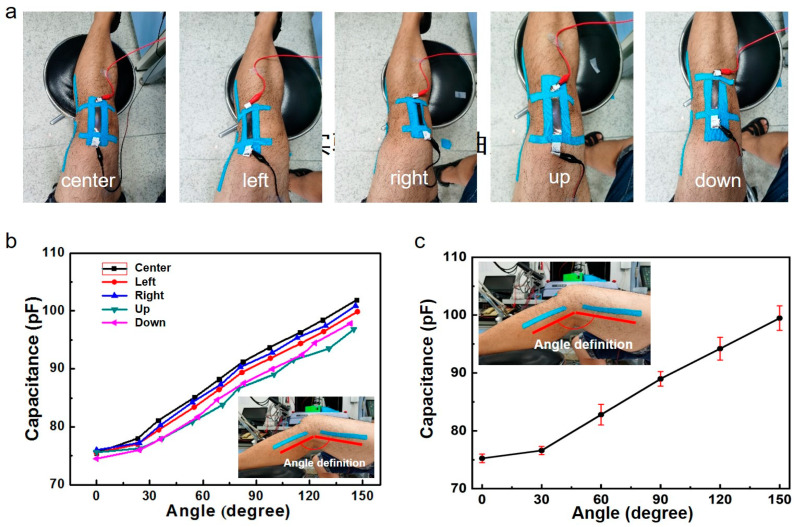
Calibration of the relationship between knee bending angle and capacitive strain sensor. (**a**) Physical images of sensors attached to different positions of the knee. (**b**,**c**) Data and error statistics of knee bending angle and sensor capacitance changes.

We further calibrated the bending angle between the sensor and the knee. As shown in Figure 4, we attached the sensor to different positions on the knee (up, down, left, right, center), collected data from the sensor, and calculated error statistics. The results show that within a certain range, the electrical signal data of our sensor attached to the knee can still accurately reflect the bending angle of the patient’s knee, indicating that the sensor is not sensitive to positional deviations attached to the knee. The micro-crack network adapts to curved surfaces (e.g., knee) by allowing independent crack relaxation at different positions. Unlike rigid crack sensors or nanowire-based sensors (positional error > 8%), our sensor achieves positional error < 3% (Figure 4b,c), which can help it be applied and promoted more quickly. In addition, in the 0–30° knee bending range (Figure 4b,c), capacitance curves overlap slightly due to minimal skin deformation: at small angles, the strain applied to the sensor is <5%, which falls near the detection limit of the capacitive response. This convergence is a result of two factors: (1) the SEBS substrate’s low modulus leads to negligible lateral deformation at small strains, and (2) the micro-cracked gold film exhibits minimal capacitance change before reaching the crack initiation threshold. However, this does not affect measurement reliability for rehabilitation applications: clinically relevant knee movements (e.g., walking, squatting) typically involve angles > 30°, where the sensor shows clear, distinguishable capacitance responses.

To quantify the accuracy of knee angle measurements across different sensor positions, we adopted a root mean square error (RMSE) model. The model is defined as follows:

$$\mathrm{RMSE}\;=\;\sqrt{\left(\theta sensor,i-\theta goniometer,i\right)*\left(\theta sensor,i-\theta goniometer,i\right)}$$

θsensor, i = knee angle measured by the dual-mode sensor (derived from capacitance–strain calibration), θgoniometer, i = reference knee angle measured by a digital goniometer (Biometrics Ltd., Newport, UK, accuracy ± 0.5°, industry standard for clinical angle measurement), N = number of data points per position (collected over of 0–150° knee flexion, 10 data points per position). Based on the capacitance–angle calibration curves and error statistics in Figure 4b,c, we calculated the RMSE for each placement position as follows:
**Sensor Placement Position****RMSE (°)**Center1.7Up2.4Down2.5Left2.2Right2.1

The “Center” position exhibits the lowest RMSE (1.7°), consistent with the tightest capacitance–angle curve clustering in Figure 4b (minimal data dispersion). Positions with slightly higher RMSEs (Up/Down/Left/Right, 2.1–2.5°) correspond to the minor capacitance curve convergence observed in the 0–30° angle range (Figure 4c), but the error remains well within clinically acceptable limits. All positions show RMSE < 2.5°, confirming that placement variations have minimal impact on measurement accuracy.

To verify measurement consistency, we conducted repeatability tests by attaching the sensor to the same knee position (central area) of three subjects, repeating 5 cycles of bending for each subject. From Figure S9, it can be seen that the measurement error of the device during repeated use is small, and the repeatability and stability performance meet the requirements. To verify the reliability and stability of the sensor, we added a set of ten cycles of tensile testing, which simulated the real situation of knee bending, and conducted controlled, large-deformation, oblique tensile testing on a tensile table. It can be seen that the tensile cycling stability of our device is very good (Figure S10). In addition, to verify the effect of bending on the device, we conducted a comparison of pure-tensile and bending-tensile composite tests on the same device. It can be seen that when the device is bent and stretched simultaneously, there is no significant difference in the capacitance change compared to pure stretching (Figure S11), depending on the mechanism of the capacitive sensor (the capacitance value is only related to the electrode area, dielectric layer thickness, and dielectric constant), and bending does not significantly change these parameters.

The special electrophysiological signal acquisition chip is used to collect the surface EMG signal of the human body, and the 555 timer is used to convert the capacitance change in the deformation sensor into the frequency change, and then the capacitance of the sensor is accurately measured by measuring the frequency. The data-receiving and -processing unit receives the collected EMG and frequency data and sends them to the WIFI wireless transmission module after unpacking, converting, packaging, and other operations (Figure S12). The human–computer interaction interface (Figures S13 and S14) receives the data sent by the WIFI module wirelessly in real time, processes the data with high-pass filtering and power–frequency notch wave, converts the capacitance value into the angle value of the human joint as required, and draws the EMG and joint angle waveform in real time. The principle of ADS front-end device testing is shown in Figure S15a; the surface EMG acquisition chip selects a low-power, eight-channel analog front-end ADS1298 dedicated to electrophysiological signal acquisition, with eight low-noise programmable gain amplifiers (PGA) and eight 24-bit high-resolution analog-to-digital converters (ADC). The data rate can be control as 250SPS to 32kSPS, with a built-in right-leg drive amplifier, lead disconnection detection, Wilson center terminal, pacing detection, and other special functions for human electrophysiological signal detection. The common mode rejection ratio (CMRR) is 115 dB, and the signal-to-noise ratio is 112 dB. The chip is widely used in medical instruments such as ECGs, EMGs, and EEGs.

**Figure 5:**
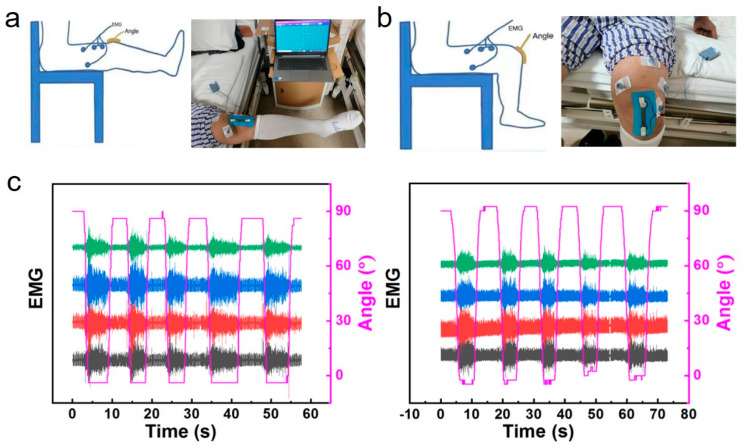
Clinical testing of knee joint electromyography and motion angle in patients with ligament injury. (**a**,**b**) The schematic of measurements of electromyography and movement angle of the knee joint in patients with ligament injury. (**c**) Corresponding data graph of EMG and knee bending angle. (Left is subject 1, right is subject 2).

In this project, the pulse-counting method is used to measure the capacitance. Its principle is to convert the capacitance into a frequency signal through the RC oscillation circuit. The capacitance value can be obtained by measuring the frequency and performing a series of operations. The principle of the LMC555 capacitance measurement circuit is shown in Figure S15b; an LMC555 chip was selected to build the RC oscillation circuit. The maximum unstable oscillation frequency can reach 3 MHz, which can accurately measure capacitance as low as 10 pF. The timing offset of the chip with temperature change is 75 ppm/°C, which has excellent temperature stability. Generally, the impact of temperature change in the application environment on the capacitance measurement accuracy is negligible. The strain and EMG dual-mode flexible sensor is applied on patients with ligament injury for real-time monitoring of knee joint movement diagnosis and rehabilitation (Figure 5a–c and Figure S16). The clinical testing cohort included two patients with unilateral anterior cruciate ligament (ACL) injury. Inclusion criteria: Confirmed ACL tear via magnetic resonance imaging (MRI) within 3 months prior to enrollment; no history of other musculoskeletal disorders (e.g., meniscal tear, osteoarthritis) or neurological diseases; ability to perform controlled knee movements (0–120° flexion) without severe pain. Exclusion criteria: Skin lesions (e.g., eczema, ulcers) at the sensor attachment site; allergic reactions to SEBS or gold; inability to comply with the testing protocol. Each patient performed standardized movements (walking, squatting, leg raising) to collect synchronous EMG and joint angle data.

**Figure 6:**
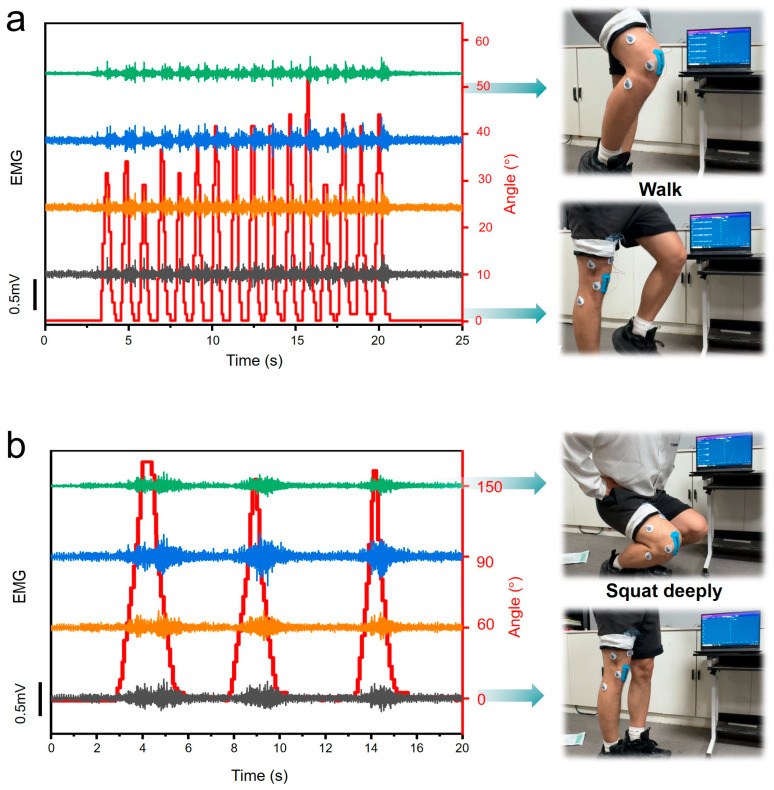
Display of leg electromyography and knee flexion angle data for subjects wearing dual-mode sensors for stationary walking (**a**) and squatting (**b**).

To rigorously demonstrate the reliability and robustness of our dual-mode acquisition device under realistic movement conditions, we conducted comprehensive performance testing across a standardized series of lower-limb motions. The evaluation protocol included four fundamental movement patterns: walking at controlled speeds (Figure 6a), deep squatting with 150° knee flexion (Figure 6b), leg raises, and running at moderate intensity, as captured in Supplementary Videos S1–S4. From the synchronized sensor outputs presented in Figure 6 and the corresponding motion sequences in Supplementary Videos S1–S4, several key performance characteristics become evident. The device maintains excellent signal acquisition fidelity during both small-amplitude movements (e.g., subtle gait adjustments) and large dynamic motions (e.g., full-range squats). The wireless transmission stability was verified, meeting the requirements for joint real-time monitoring.

The acquired dual-mode data streams enable clear, quantitative interpretation of the subject’s movement state. Through simultaneous visualization of the EMG amplitude (0–500 μV range) and knee joint angle (0–150° range), it is remarkably straightforward to determine both the muscular effort level and precise kinematic position being exerted by the monitored subject at any movement phase. This comprehensive evaluation confirms that the dual-mode device operates with exceptional stability and reliability across diverse movement conditions. The strain sensors maintain measurement consistency (<3% variation) despite significant skin deformation during dynamic motions, while the EMG electrodes demonstrate excellent motion artifact rejection through their optimized skin-contact design. Existing wearable systems either measure only joint angle [3,4] or EMG [10,13], missing the synergy between muscle activation and kinematics. Our dual-mode fusion improves motion decoding accuracy. For similar joint angles (e.g., 90° knee flexion during walking vs. squatting), the EMG amplitude discriminates muscle effort (walking: 100–200 μV; squatting: 300–500 μV, Figure 6), enabling classification of movement types with higher accuracy. The combined results from these controlled movement trials and Supplementary Videos S1–S4 evidence collectively establish our device as a robust solution for practical joint monitoring applications requiring synchronized mechanical and electrophysiological data acquisition. Apart from this, we tested the sensor’s capacitance at 10–50 °C under 0%, 30%, and 50% strain (Figure S17). For all strain levels, capacitance shows minimal variation (total change < 2.5%) across the full temperature range, confirming the sensor’s low temperature coefficient and environmental robustness. At each temperature, capacitance increases with strain (consistent with the sensor’s capacitive sensing mechanism, where higher strain expands the electrode area and reduces dielectric thickness). In the physiological temperature range (32–38 °C, typical for human activity), capacitance fluctuations are <0.7% for all strains, validating the sensor’s reliability for wearable joint monitoring applications.

We compare our sensor with state-of-the-art stretchable strain sensors and dual-mode systems.

From Table 1, our sensor balances key performance metrics for practical wearable applications; while resistive sensors [3,5,34] offer higher GF, they suffer from higher hysteresis and drift. Our capacitive design achieves low hysteresis (<2%), positional insensitivity (<3% error), and seamless EMG integration—critical for reliable joint monitoring. Compared to the only reported capacitive dual-mode system [9], our work offers higher stretchability, lower hysteresis, and EMG (vs. ECG) integration, which is more relevant for muscle–joint motion analysis.

In order to make EMG data more abundant and accurate, we have further developed a multi-channel (64 channels) flexible stretchable electrode. It was applied to a person’s forearm for preliminary testing, and the test data is shown in Figures S18 and S19. In the future, we will replace commercial single-channel electrodes with multi-channel thin-film stretchable electrodes to fuse with strain sensors in a dual-modal manner, thereby promoting more accurate joint monitoring.

## 4. Conclusions

In this article, we designed and implemented a wireless transmission device platform for dual-mode monitoring of EMG signals and knee angle. By combining these two monitoring modes, we have achieved more comprehensive and accurate monitoring and analysis of joint movement status. EMG signals provide detailed information about muscle contraction and relaxation, while knee angle can reflect joint movement trajectory and stability. Through wireless transmission technology and designed corresponding software, we have achieved real-time data transmission and analysis, allowing doctors to receive quantitative monitoring and give helpful guidance during the rehabilitation process for patients. This flexible sensor not only improves the accuracy of monitoring in rehabilitation therapy, but also brings new possibilities for applications in other fields such as sports training. In the future, we can further optimize the sensor accuracy and data-processing algorithms of the equipment to improve monitoring accuracy and real-time performance. In addition, by combining machine learning and artificial intelligence technologies, we can develop more intelligent monitoring systems that provide personalized data analysis and recommendations based on individual characteristics and movement patterns. These will further promote the application and development of wireless transmission, multimodal surface flexible sensors in the fields of health management and exercise rehabilitation.

## Figures and Tables

**Table 1:** Performance benchmarking with existing technologies.

Parameter	Our Work	[3] Graphene Sensor	[5] Crack-Based Sensor	[34] Ag Nanowire Sensor	[9] Capacitive Dual-Mode
Stretchability	>80%	50%	100%	300%	60%
Gauge Factor (GF)	0.83 (capacitive)	30 (resistive)	500 (resistive)	20 (resistive)	0.8 (capacitive)
Hysteresis	<2%	8%	15%	5%	4%
Response Time	70–100 ms	200 ms	50 ms	150 ms	120 ms
Detection Limit	0.1% strain	0.5% strain	0.01% strain	0.2% strain	0.3% strain
Positional Insensitivity	<3% error	12% error	15% error	8% error	6% error
Dual-Mode Integration	Strain+ EMG	Strain-only	Strain-only	Strain-only	Strain + ECG

Note: References correspond to the manuscript’s reference list. GF for capacitive sensors is defined as (ΔC/C_0_)/ε, where ε is strain.

## Data Availability

The raw data supporting the conclusions of this article will be made available by the corresponding author on request.

## Supplementary Materials

The following supporting information can be downloaded at: https://www.mdpi.com/article/10.3390/mi17010077/s1, Figure S1. The overall plan of the equipment. Figure S2. (a) Physical image of strain sensor (b): Optical mirror image of the thickness at each position after back-to-back self-adhesive bonding. Figure S3. Physical detail diagram of capacitive strain sensor. Figure S4. Thickness data of sensors from different batches. Figure S5. Microscopic morphology of electrode surface. Figure S6. SEM images of SEBS gold films prepared from different batches. Figure S7. SEM images of SEBS gold films prepared from different batches. Figure S8. (a) The capacitance change in strain sensors during a one-week aging test at 60 °C. (b)Tensile cycling test of the device at a high temperature of 50 degrees. Figure S9. Repeated tensile stability testing of the same strain sensor on different subjects for five cycles. Figure S10. Oblique tensile cyclic testing of strain sensors on a tensile table. Figure S11. (a) Strain sensor tensile testing. (b) Strain sensor tensile and bending composite testing. Figure S12. Dual-mode monitoring experiment of electromyography and bending angle, and the physical picture of the wireless receiver transmitter. Figure S13. Strain sensor software angle calibration interface and performance testing. Figure S14. Screenshot of the interface of the dual-mode detection software for the electromyographic and bending angle. Figure S15. Hardware schematic. Figure S16. Patient electromyography and bending angle dual-mode monitoring test. Figure S17. Capacitance stability of the capacitive strain sensor under different strains across 10–50 °C. Figure S18. Physical image of multi-channel stretchable thin film electrode array attached to forearm for testing electromyographic signals and corresponding 64-channel data graph. Figure S19. EMG and time-frequency data of 4 out of 64 channels. Video S1: Stability of collecting stationary walking signals using a dual-mode wireless transmission device with strain sensors and four-channel electromyography monitoring; Video S2: Stability display of electromyographic and knee angle signal output after leg lifting and rest; Video S3: Display of output stability of dual-mode signals during the transition from slow walking to jogging; Video S4: Display of bimodal signal output during the period from minimum to maximum knee angle.
