# Supplementary material for: Strain and Electromyography Dual-Mode Stretchable Sensor for Real-Time Monitoring of Joint Movement
Source: Micromachines (Basel). 2026 Jan 6;17(1):77. doi: 10.3390/mi17010077 (PMC12843643; doi:10.3390/mi17010077)
Supplement: Supplementary file 1 [file micromachines-17-00077-s001.zip › micromachines-4047778-supplementary.pdf]

Supporting Information

Strain and electromyography dual-mode flexible sensor for real-time monitoring of joint movement

Hanfei L[3], Xiaomeng Zhou, Shouwei Yue, Qiong Tian, Qingsong Li, Jianhong Gong, Yong Yang, Fei Han, Hui Wei, Zhiyuan Liu, Yang Zhao

This file includes:

Figures S1 to S19

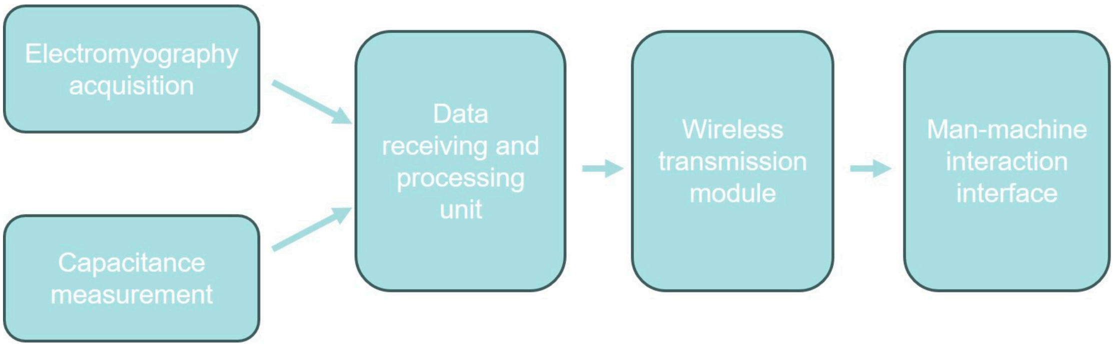

Figure S1. The overall plan of the equipment.

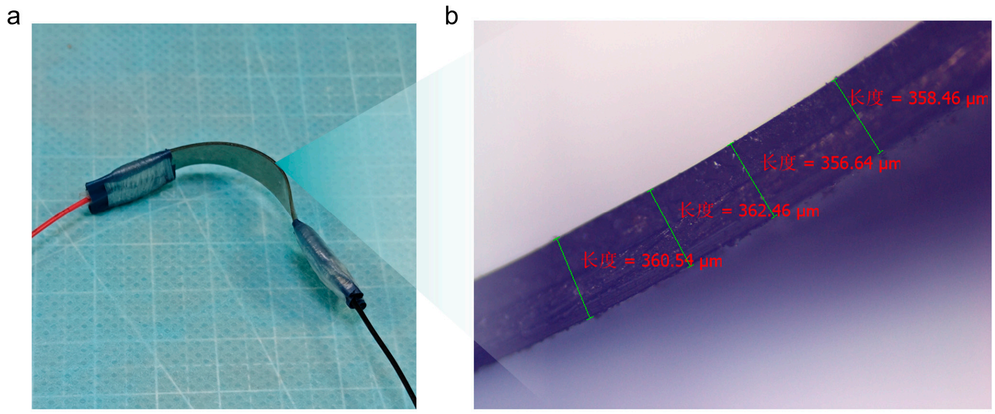

Figure S2. (a) Physical image of strain sensor (b): Optical mirror image of the thickness at each position after back-to-back self-adhesive bonding.

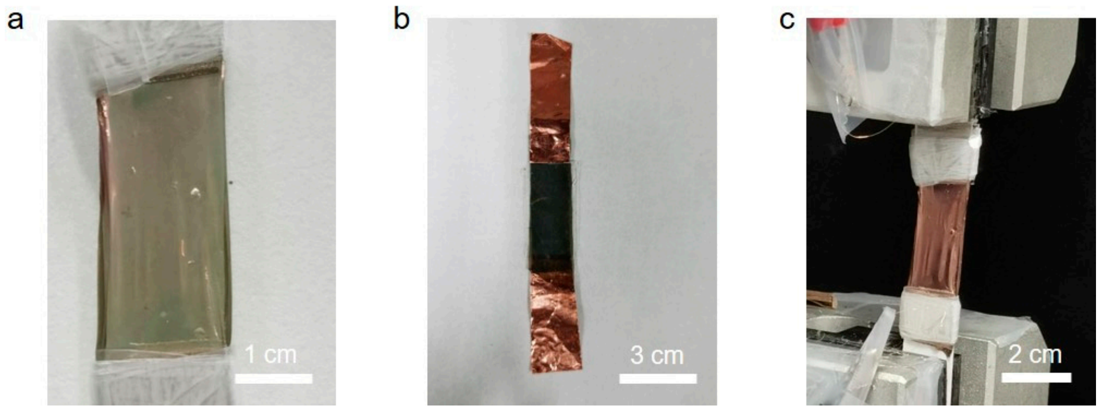

Figure S3. Physical detail diagram of capacitive strain sensor.

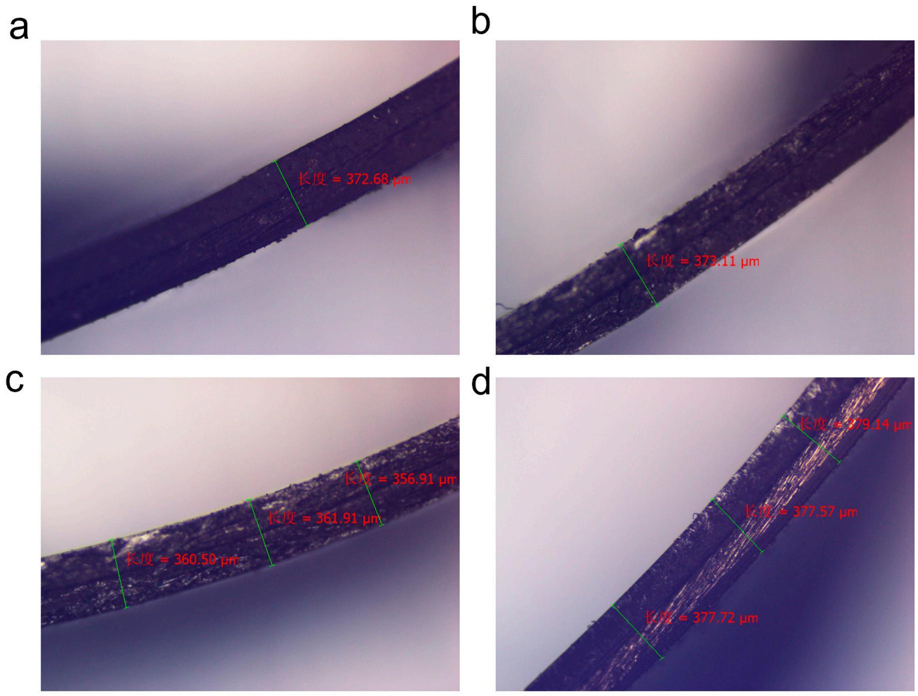

Figure S4. Thickness data of sensors from different batches.

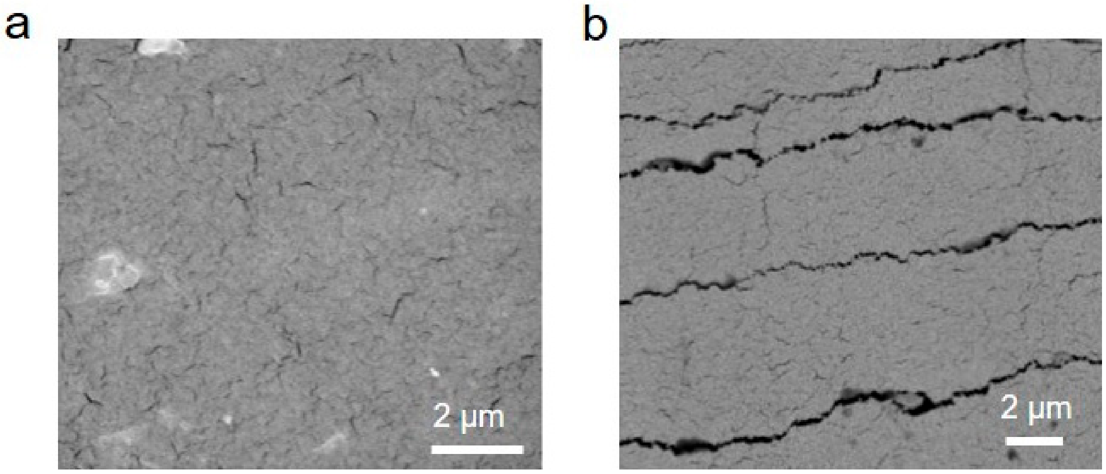

Figure S5. Microscopic morphology of electrode surface. (a) Initial morphology. (b) Morphology during stretching.

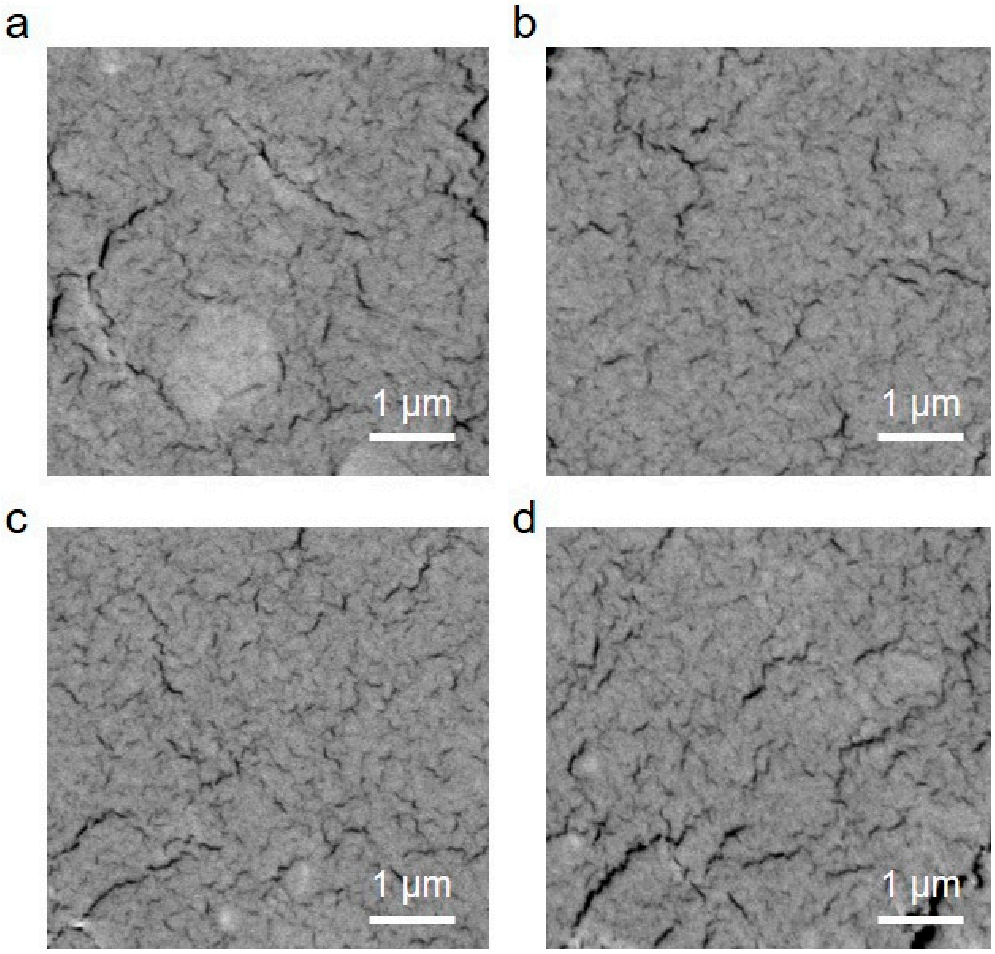

Figure S6. SEM images of SEBS gold films prepared from different batches.

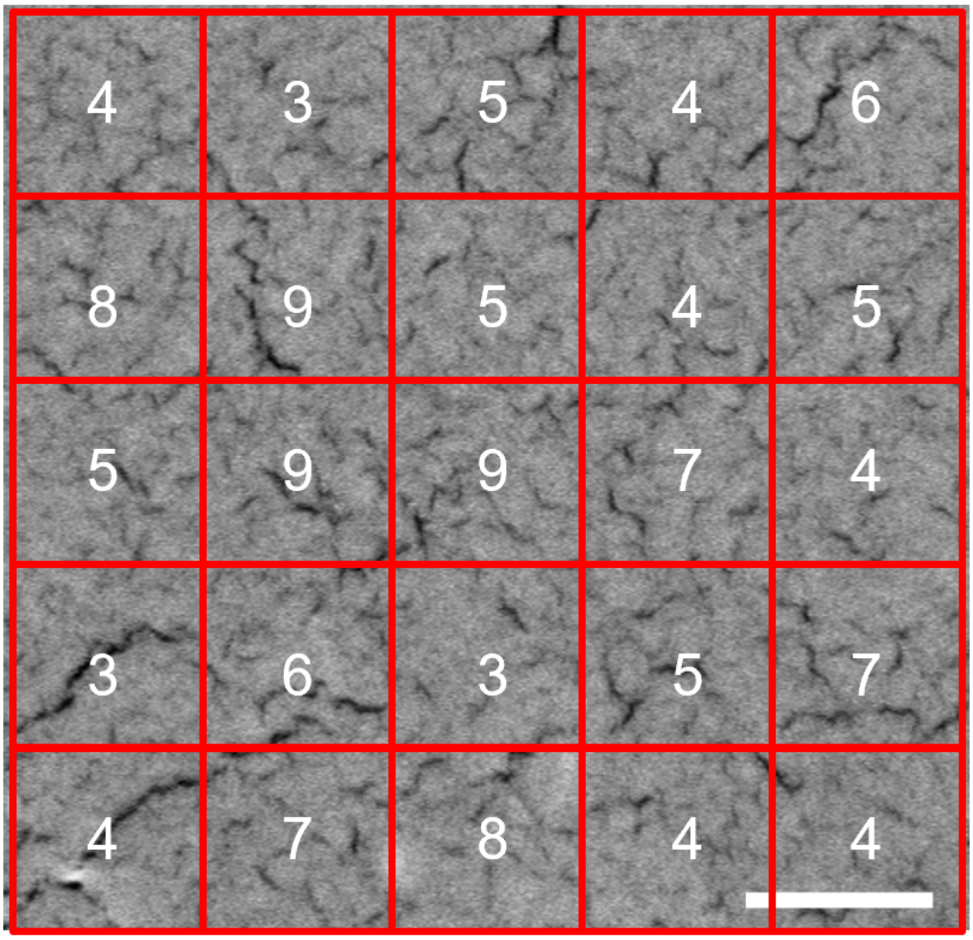

Figure S7. SEM images of SEBS gold films prepared from different batches.

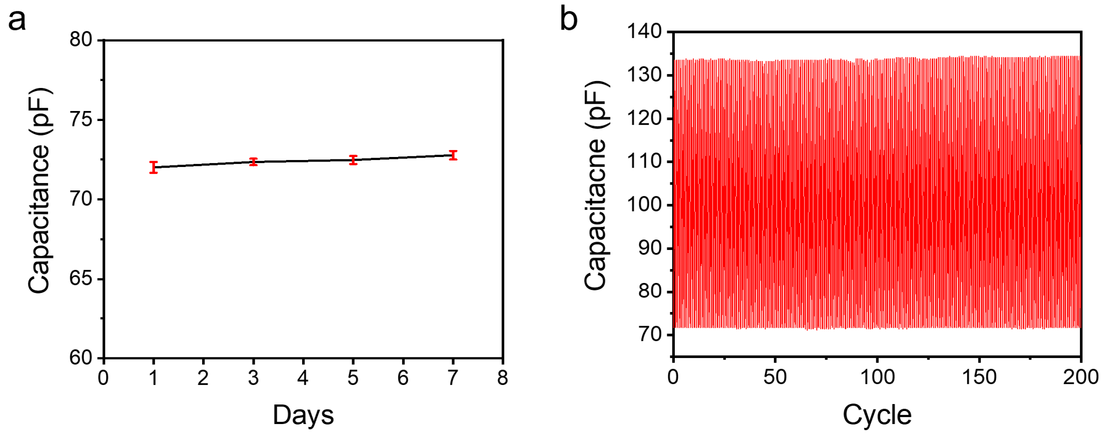

Figure S8. (a) The capacitance change of strain sensors during a one week aging test at 60°C. (b)Tensile cycling test of the device at a high temperature of 50 degrees.

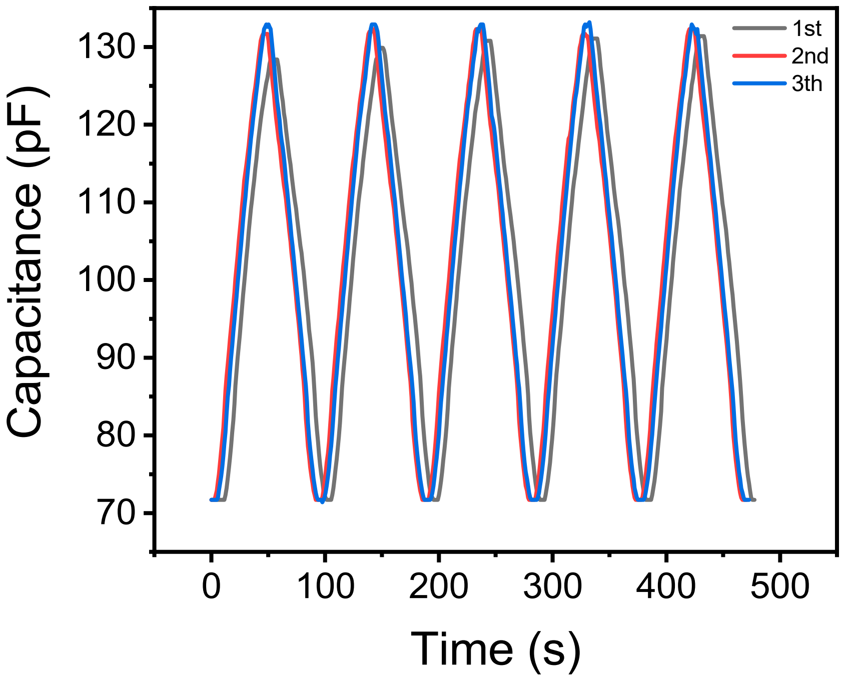

Figure S9. Repeated tensile stability testing of the same strain sensor on different subjects for five cycles.

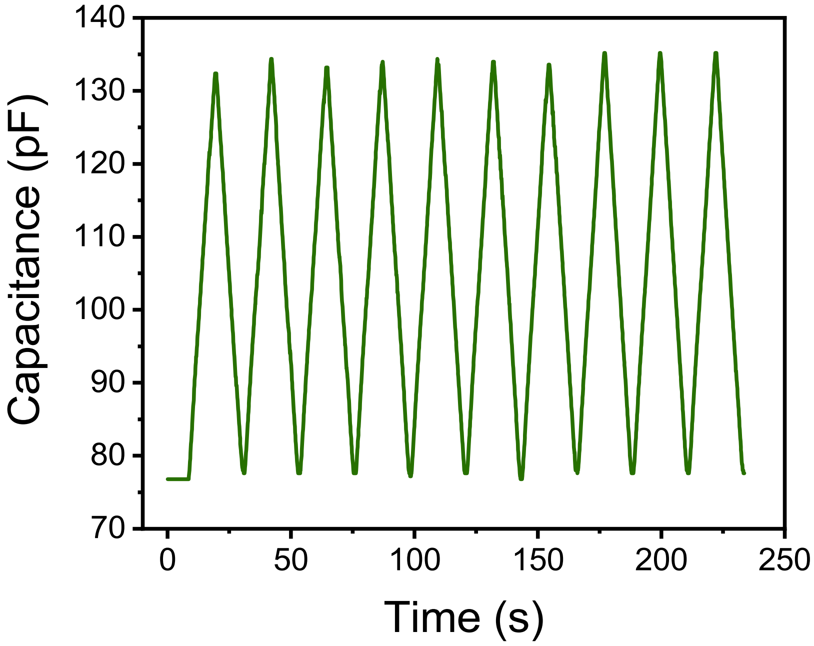

Figure S10. Oblique tensile cyclic testing of strain sensors on a tensile table.

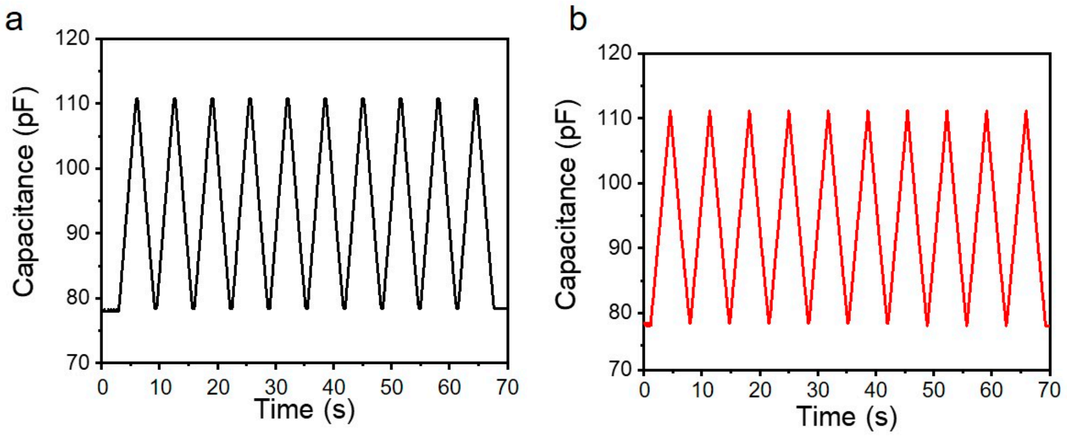

Figure S11. (a) Strain sensor tensile testing. (b) Strain sensor tensile and bending composite testing.

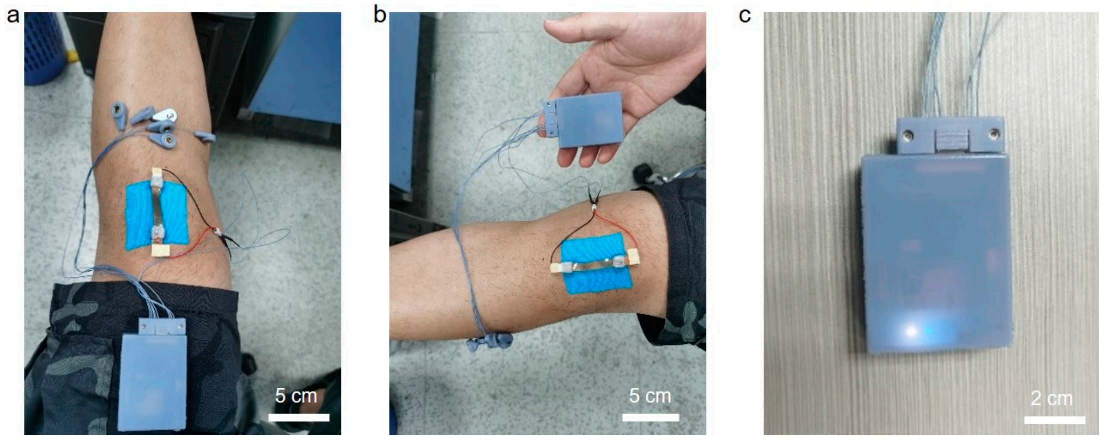

Figure S12. Dual mode monitoring experiment of electromyography and bending angle and the physical picture of the wireless receiver transmitter.

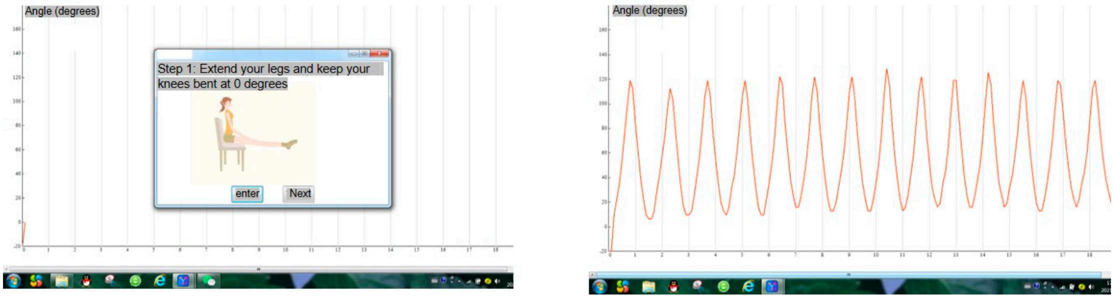

Figure S13. Strain sensor software angle calibration interface and performance testing.

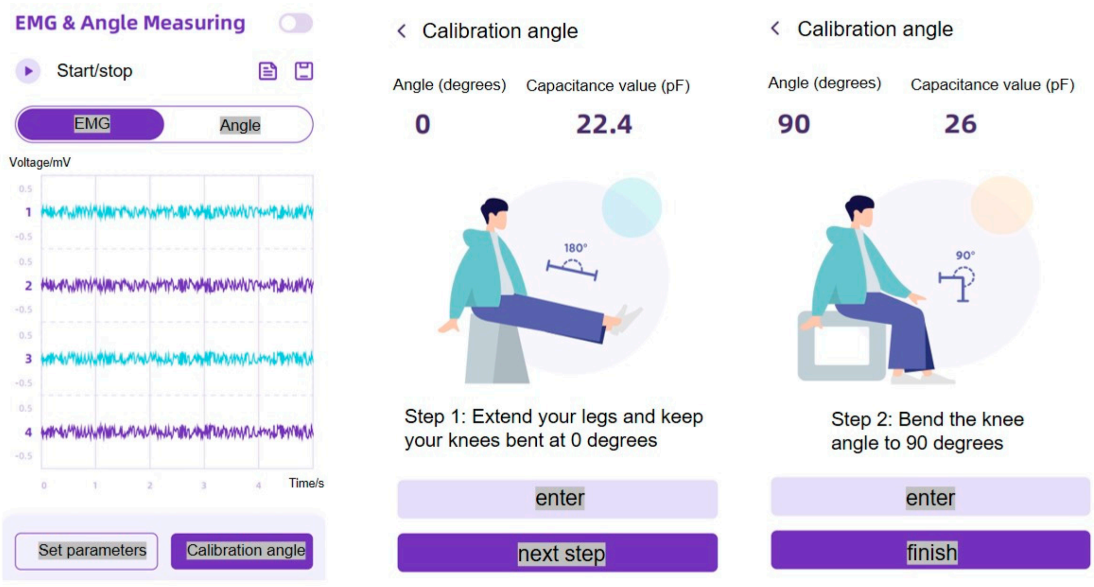

Figure S14. Screenshot of the interface of the dual-mode detection software for electromyographic and bending angle.

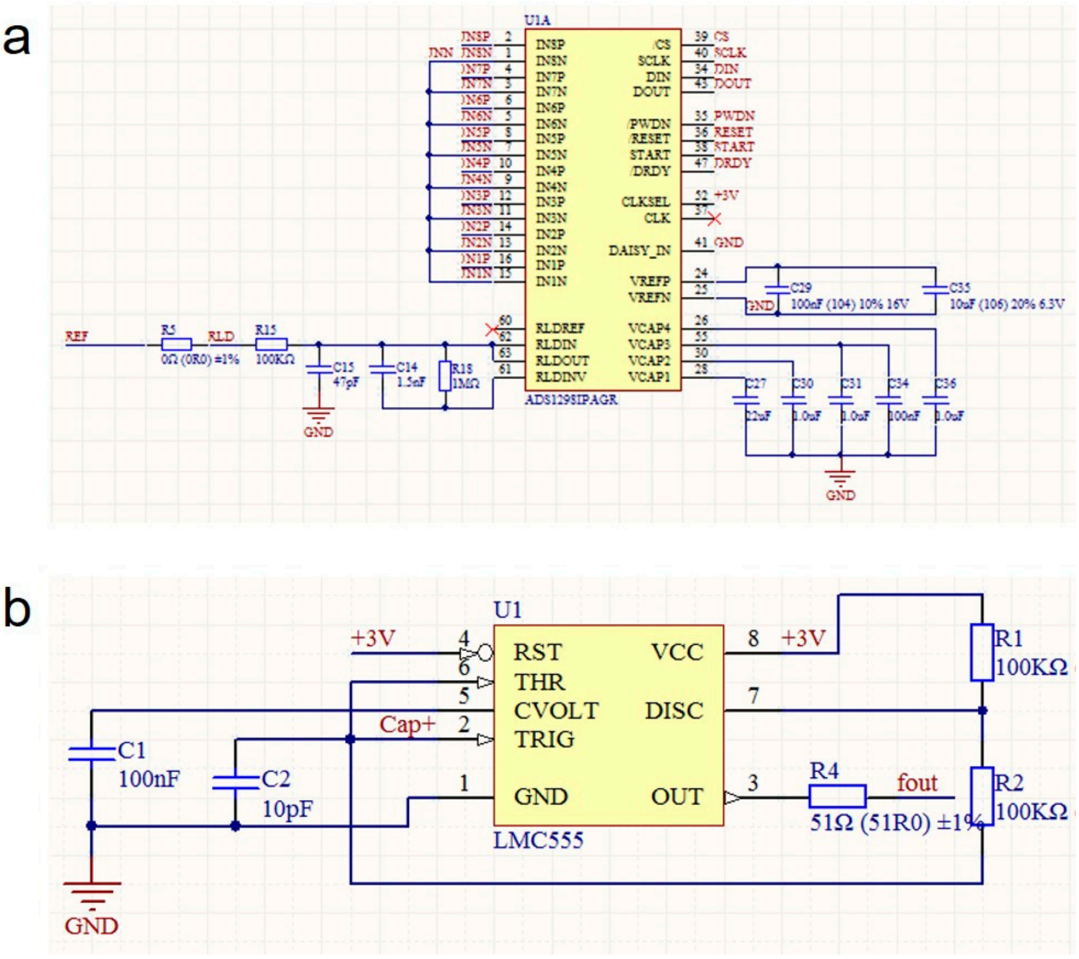

Figure S15. Hardware schematic. (a) Principle of ADS1298 EMG measurement front end (b) Principle of LMC555 capacitance measurement circuit.

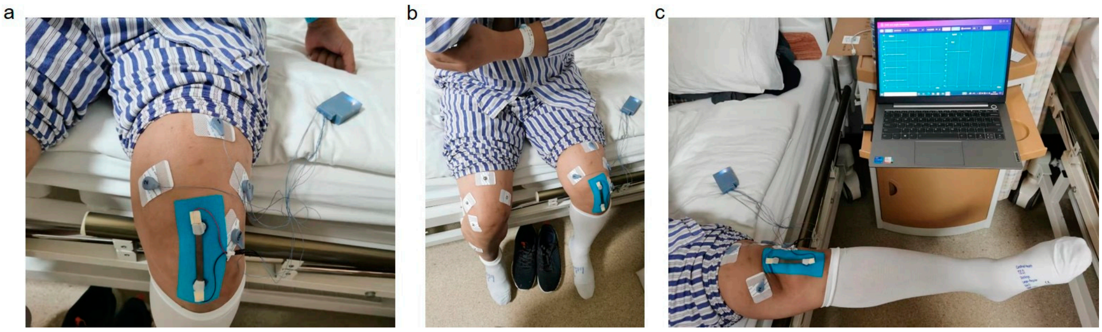

Figure S16. Patient electromyography and bending angle dual-mode monitoring test.

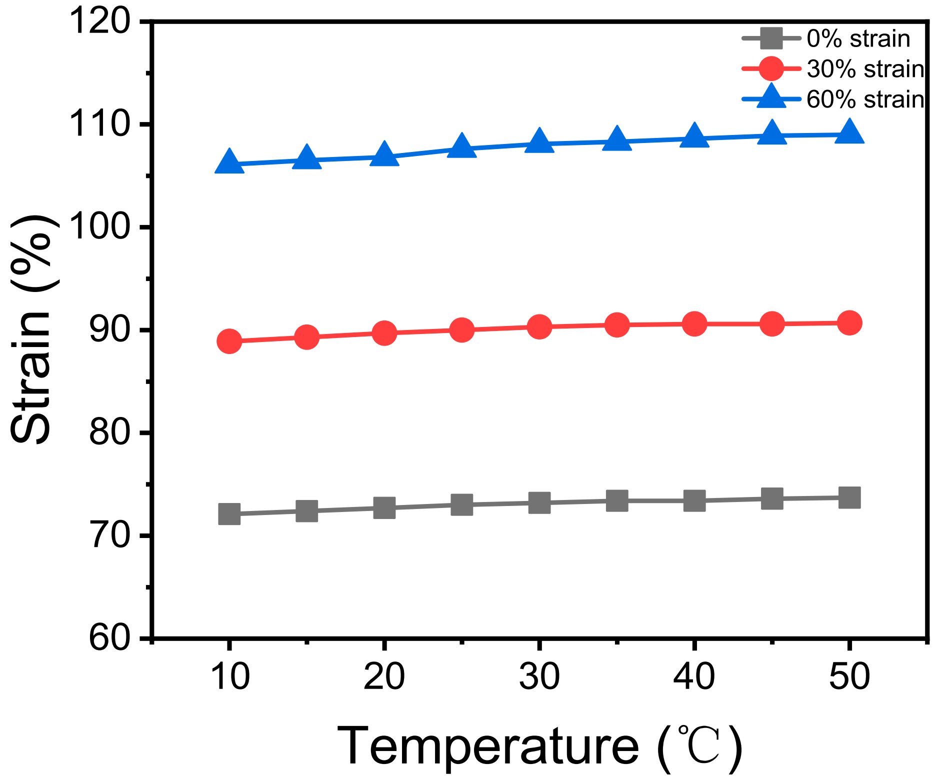

Figure S17. Capacitance stability of the capacitive strain sensor under different strains across 10–50°C.

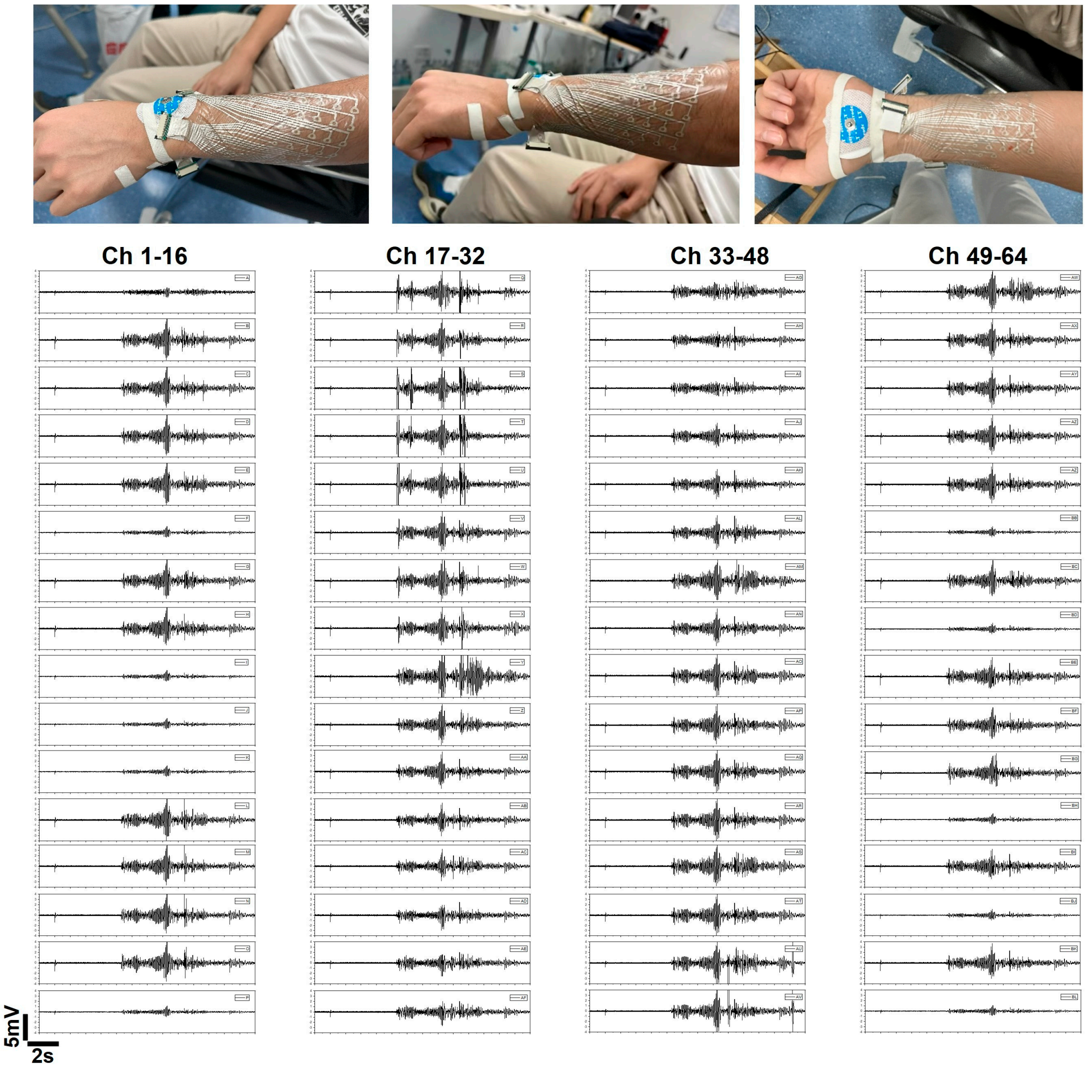

Figure S18. Physical image of multi-channel stretchable thin film electrode array attached to forearm for testing electromyographic signals and corresponding 64 channel data graph.

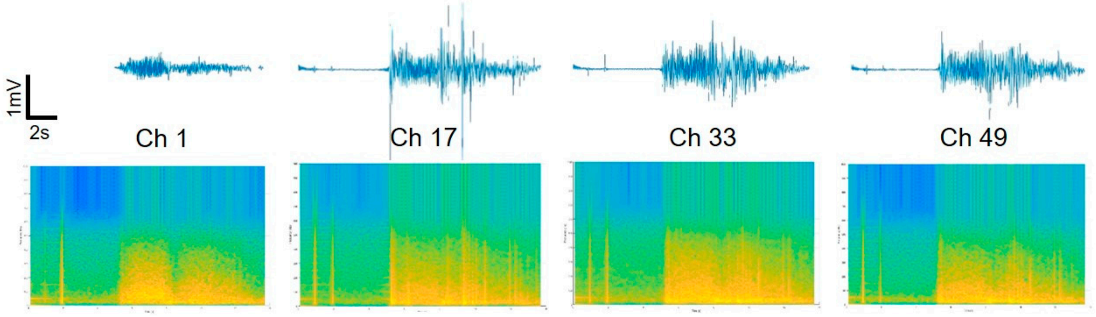

Figure S19. EMG and time-frequency data of four out of 64 channels.
